# Effect of Pre-Damage on the Behavior of Axially and Eccentrically Compressed Concrete Cylinders Confined with PBO-FRCM

**DOI:** 10.3390/ma18122881

**Published:** 2025-06-18

**Authors:** Maciej Pazdan, Tomasz Trapko, Michał Musiał

**Affiliations:** Department of Building Structures, Faculty of Civil Engineering, Wrocław University of Science and Technology, 27 Wybrzeże Stanisława Wyspiańskiego st., 50-370 Wrocław, Poland; tomasz.trapko@pwr.edu.pl (T.T.); michal.musial@pwr.edu.pl (M.M.)

**Keywords:** strengthening, composite, FRCM, PBO, concrete, compression

## Abstract

In the case of strengthening building structures, the process usually involves elements that have a certain loading history and are typically subjected to loading during the strengthening process. In scientific research, on the other hand, strengthening is usually applied to elements that are not representative of real structures. This article presents a study of the effect of pre-damage on the behavior of eccentrically compressed concrete cylinders confined with PBO-FRCM (fabric-reinforced cementitious matrix with PBO fibers) composite. Concrete confinement introduces a favorable triaxial stress state, which leads to an increase in the compressive strength of concrete. FRCM systems are an alternative to FRP (fiber-reinforced polymer) composites. Replacing the polymer matrix with a mineral matrix primarily improves the fire resistance of the strengthening system. The elements were made of concrete with a compressive strength of about 40 MPa, which is typical for current reinforced concrete columns. Pre-damage was induced by loading the test elements to 80% of the average compressive strength and then fully unloading. The elements were then strengthened with three layers of PBO-FRCM composite and subjected to axial or eccentric compression with force applied at two different eccentricities. In addition to electric strain gauges, a digital image correlation system was used for measurements, to identify the initiation of PBO mesh overlap delamination. This study analyzed the elements in terms of load-bearing capacity, deformability, ductility, and failure mechanisms. In general, there was no negative effect of pre-damage on the behavior of the tested elements.

## 1. Introduction

Strengthening of compressed reinforced concrete elements can be achieved by enlarging the cross-section or by confining the concrete. Traditional strengthening methods include, for example, reinforced concrete jackets [1,2], steel jackets [3], or steel jackets combined with concrete or mortar injection [4]. Among modern techniques utilizing composite materials such as FRP (fiber-reinforced polymer) or FRCM (fabric-reinforced cementitious matrix), strengthening is achieved primarily through concrete confinement. Composite strengthening systems have significantly smaller dimensions due to the use of high-strength fibers. FRCM composites, in which the matrix is a mineral-based mortar, offer higher resistance to elevated temperatures compared to conventional FRP composites [5,6,7].

There are numerous studies on strengthening concrete compression elements with FRCM composites, mainly with PBO-FRCM (fabric-reinforced cementitious matrix with PBO fibers). These studies usually concern axial compression [5,6,7,8,9,10,11,12], and in the case of slender reinforced elements (usually of rectangular cross-section), also eccentric compression [13,14,15]. The behavior of confined elements under eccentric compression is also often investigated with reference to CFRP (carbon fiber-reinforced polymer) composites [16,17,18]. Research on this topic is usually conducted on not previously loaded elements; however, elements eligible for strengthening usually have some load history, which can affect their behavior after strengthening. The number of publications describing the study of this phenomenon, especially for PBO-FRCM composites, is relatively limited.

It is important to distinguish between two issues related to the load history of the elements under study. The first is the pre-load effect, which concerns elements that are strengthened under load [19] (usually for FRP composites), and the second is the pre-damage effect, which concerns elements that are pre-damaged—usually as a result of loading to a certain level of stress [20,21]. These effects lead to different results, as they have different effects on material degradation and deformability after strengthening application. This article focuses on the pre-damage effect.

The authors of [19] presented a pre-load effect study conducted on cylindrical concrete specimens (diameter of 150 mm and height of 300 mm) that were pre-loaded (20%, 50%, and 80% of failure load) and strengthened with CFRP composite. It was shown that pre-loading has a negative effect on load-bearing capacity, ultimate longitudinal strain, and Young’s modulus, as it leads to the formation of microcracks in the concrete, which weaken its structure.

In [20], a study was presented on the effect of pre-damage on axially compressed reinforced concrete columns (diameter of 200 mm and height of 800 mm) with circular cross-sections and their behavior after strengthening with PBO-FRCM composite. Pre-damage was induced by loading the columns once to load-bearing capacity or loading them three times to 75% of load-bearing capacity. The number of composite layers used, and the spacing of stirrups, were varied in the test elements, which had a greater effect on the behavior of the test columns than the levels of pre-damage. The strengthening reinforcement ratio determined the increase in load-bearing capacity, while the spacing of stirrups determined the failure mechanism. The effect of loading the specimens once to the failure force level was negligible from the standpoint of load-bearing capacity. Smaller increases in load-bearing capacity after strengthening were recorded for elements subjected to three loading cycles up to 75% of load-bearing capacity.

Article [21] describes tests analogous to those in [20]; however, reinforced concrete columns with a square cross-section (side length of 180 mm and height of 800 mm) were used. The specimens were subjected to the same pre-damage procedures and were divided into the same groups as in [20]. However, due to the different shape of the cross-section, which affects the distribution of confinement pressure, slightly different results were obtained. In this case, the number of composite layers had a decisive influence on both the load-bearing capacity and failure mechanism. Both loading up to 100% of the load-bearing capacity and loading up to 75% of the load-bearing capacity in three cycles influenced the strengthening ratios. The effect of pre-damage on the elements and their subsequent behavior was negligible compared to the effect of the number of composite layers and stirrup spacing.

The referenced studies concern axially compressed elements, mainly with steel reinforcement. Moreover, in both [20,21], the elements were made of concrete with a compressive strength of approximately 20 MPa. This article, on the other hand, deals with tests conducted on specimens compressed both axially and eccentrically, without reinforcement (to isolate the effect of the composite on the behavior of the tested elements), made of concrete with a strength of approximately 40 MPa—typical for currently designed reinforced concrete columns. Concrete with higher strength has more brittle behavior, which affects the effectiveness of the composite confinement [22].

## 2. Materials and Methods

### 2.1. Preparation of Concrete Cylinders

All 27 cylinder specimens, with a diameter of 150 mm and a height of 300 mm, were prepared based on the concrete mix proportions presented in Table 1. The average cylinder compressive strength of the concrete was f_cm0_ = 39.10 MPa (CV = 1.1%). Concrete curing was carried out at a temperature of approximately 20 °C on a grate over the surface of water, for 28 days.

### 2.2. Strengthening of the Concrete Cylinders

**Figure 1:**
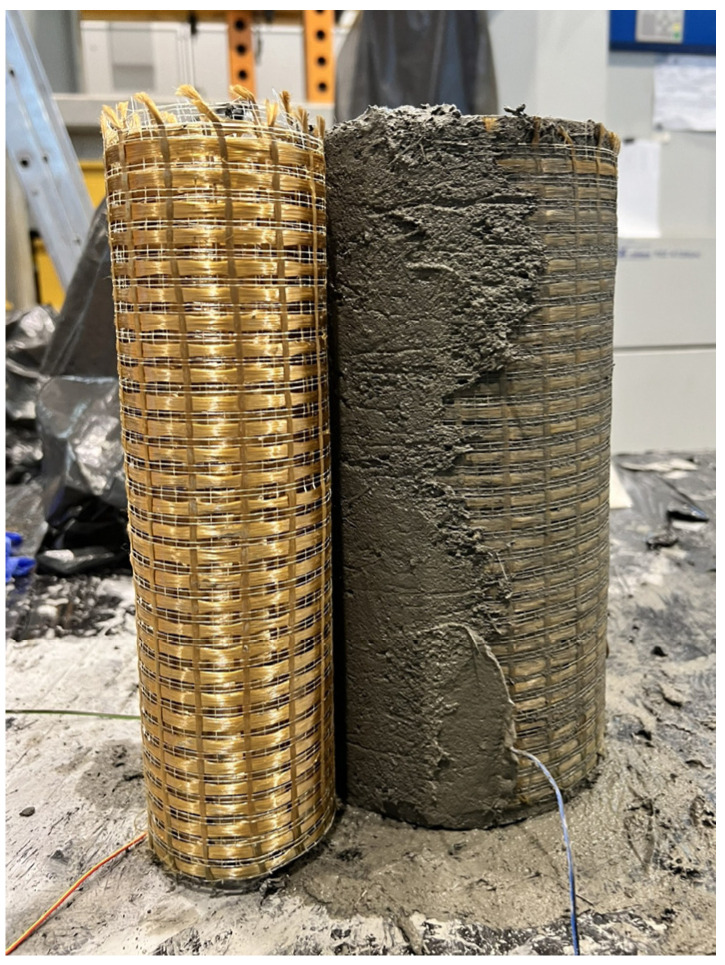
Application of PBO-FRCM system.

The strengthening system applied to the cylinders was a PBO-FRCM system, consisting of bidirectional mesh PBO-MESH 70/18 and modified cementitious mortar MX-PBO Concrete, the parameters of which are shown in Table 2 [23]. Figure 1 shows the application of the PBO-FRCM system. All strengthened specimens were confined with three layers of PBO mesh with an overlap of 1/4 of the cylinder circumference. Each specimen was wrapped three times with a single long sheet of PBO mesh. The application of each mesh layer was preceded by the application of a mortar layer. The final layer of the mesh was also covered with a layer of mortar.

### 2.3. Preparation of the Specimens for Compression Tests

After the concrete curing process was completed, the electric strain gauges were installed on the surface of the concrete. Strain gauges were also installed on the composite, in the case of strengthened specimens. Table 3 shows the arrangement of the strain gauges for all types of specimens.

**Figure 2:**
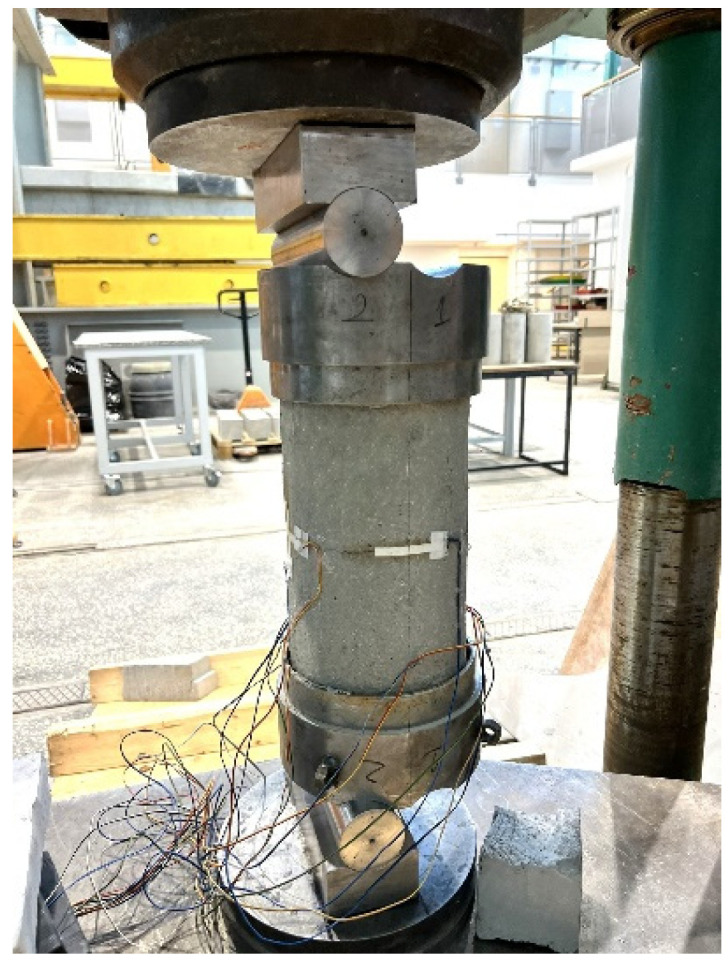
Specimen during eccentric compression test.

Eccentric compression was carried out using rigid steel caps with hinges, glued to the loaded surfaces of the cylinders with epoxy adhesive. Figure 2 shows an element during an eccentric compression test.

A testing machine with a capacity of 2000 kN was used in the tests. The loading rate was approximately 10.5 kN/s, which corresponds to a stress increase of approximately 0.6 MPa/s in the case of axially compressed specimens.

### 2.4. Pre-Damage Procedure

**Figure 3:**
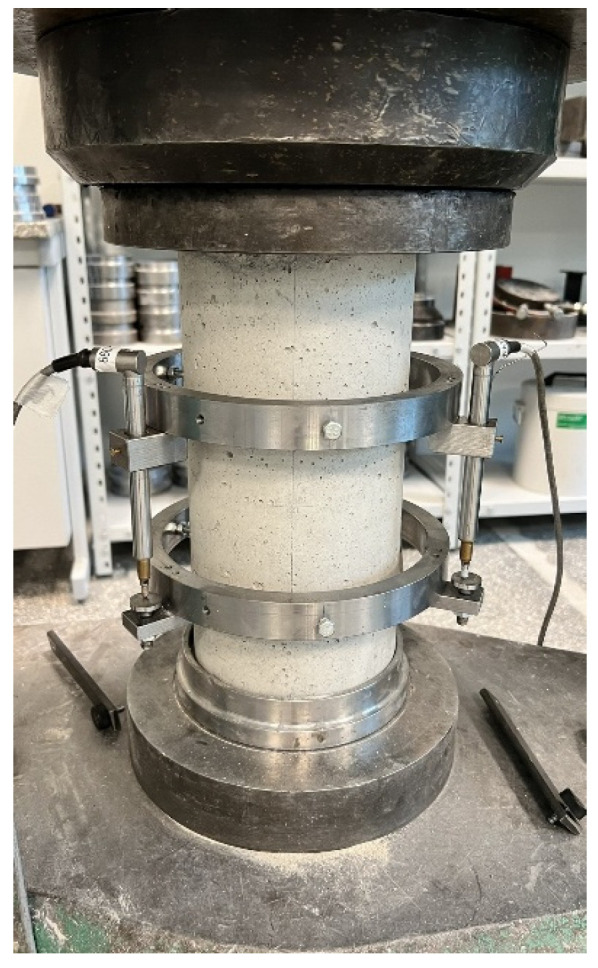
Specimen during the pre-damage process.

Nine specimens were subjected to the pre-damage process by axial loading prior to the application of the composite. The cylinders were loaded once, monotonically, to a level of 80% of the average failure force determined from the reference specimens. The force was maintained for a period of 30 s, after which the elements were unloaded. This process was carried out before strain gauges were applied; therefore, the axiality of force application was checked using a compressometer with three LVDTs spaced every 120°. Figure 3 shows a specimen with the compressometer during the pre-damage process.

## 3. Results

The test specimens were analyzed in terms of failure mechanisms, load-bearing capacity, deformability, and ductility. The failure mechanism of axially compressed specimens was also analyzed in terms of crack development at the end of the PBO mesh overlap, using the DIC system. The results of the tests are reported in Table 4. The lack of some results (“-”) is due to the failure of strain gauges before the end of the test.

### 3.1. Failure Modes

#### 3.1.1. Axially Compressed Specimens

**Figure 4:**
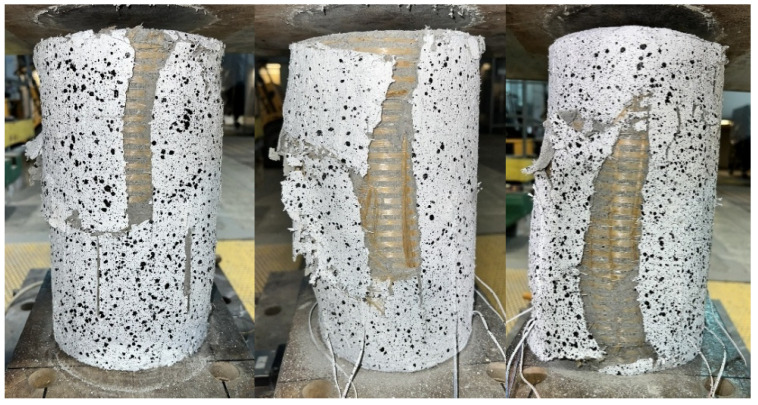
Failure modes of axially compressed confined specimens, not pre-damaged.

**Figure 5:**
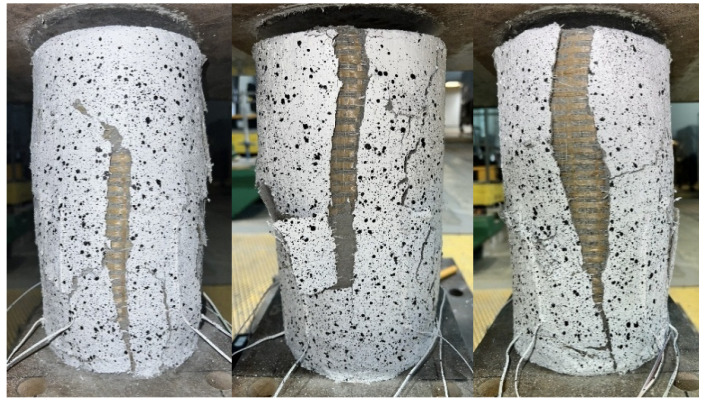
Failure modes of axially compressed confined specimens, pre-damaged.

Failure of all strengthened specimens subjected to axial compression testing was due to delamination of the PBO mesh overlap (at the fabric–matrix interface), and partial rupture of the hoop PBO fibers. In the case of pre-damaged specimens, the predominance of overlap delamination was observed. Figure 4 and Figure 5 show images of the failure of strengthened axially compressed specimens.

**Figure 6:**
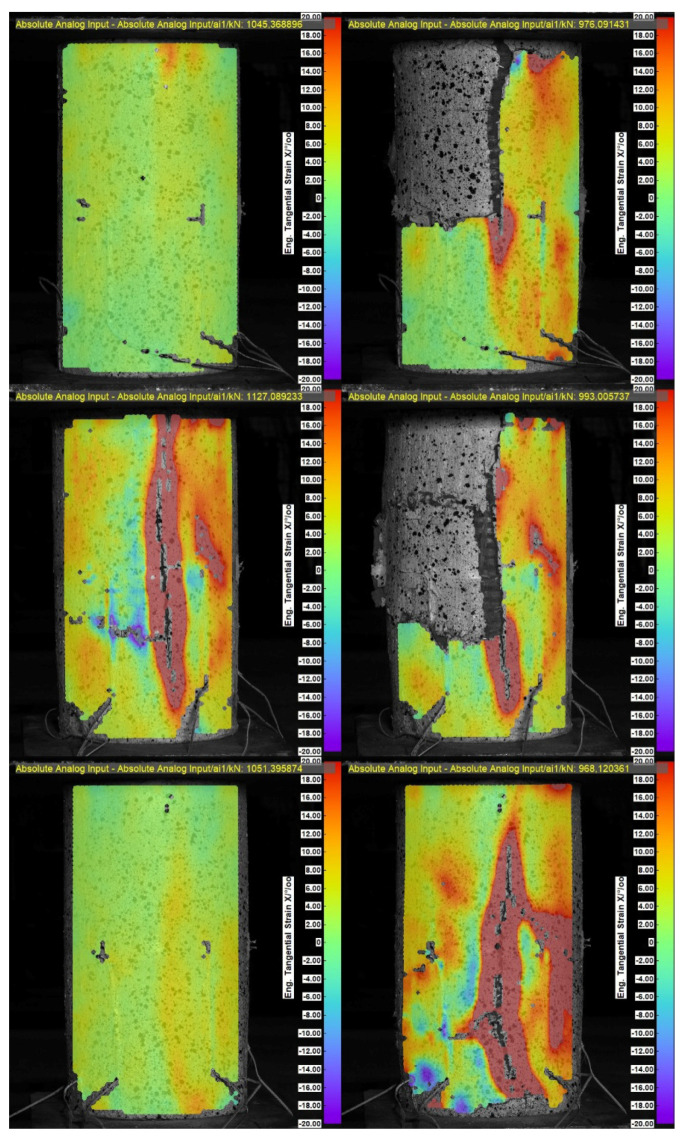
Hoop strain in the PBO mesh overlap zone—for specimens that were not pre-damaged. Illustrations on the left—at failure force; illustrations on the right—post-failure.

**Figure 7:**
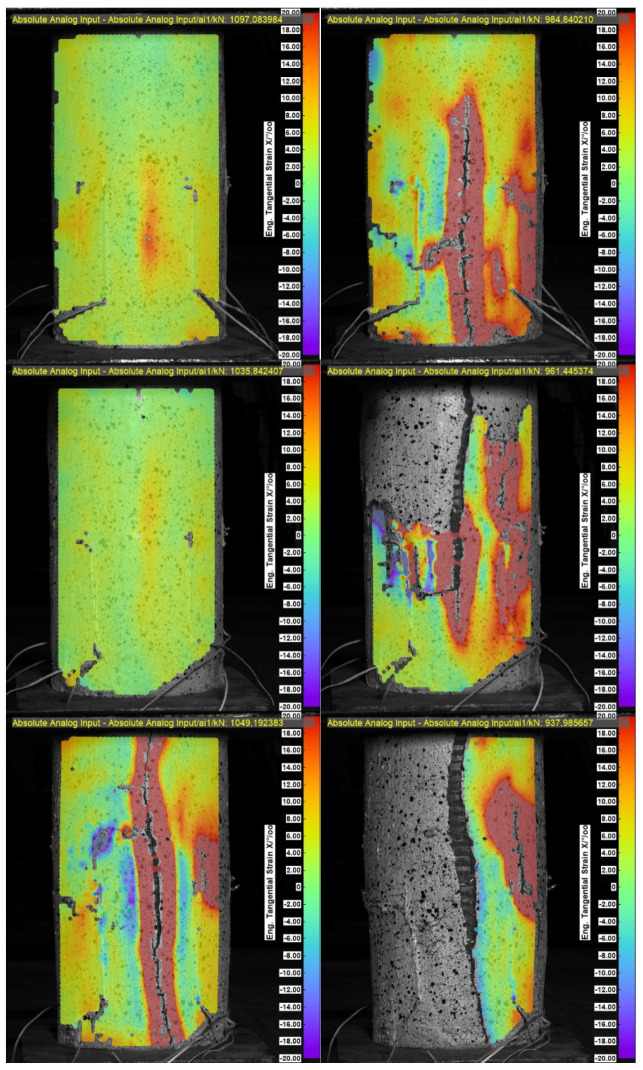
Hoop strain in the PBO mesh overlap zone—for pre-damaged specimens. Illustrations on the left—at failure force; illustrations on the right—post-failure.

The failure of axially compressed elements was also analyzed using the DIC system. The analysis was performed using hoop strain maps (for qualitative assessment of the phenomenon) and measurements of the development of crack width at the end of the PBO mesh overlap, as shown in Figure 6 and Figure 7.

The failures of both specimens that were not pre-damaged and pre-damaged specimens followed a similar pattern (Figure 6 and Figure 7). When the specimens reached stresses close to the compressive strength, cracks were noted at the ends of the PBO mesh overlaps; however, in most cases (except for two specimens), they had a relatively small width—Figure 6 and Figure 7—left. This was followed by sudden delamination of the composite and a decrease in longitudinal stress—Figure 6 and Figure 7—right.

**Figure 8:**
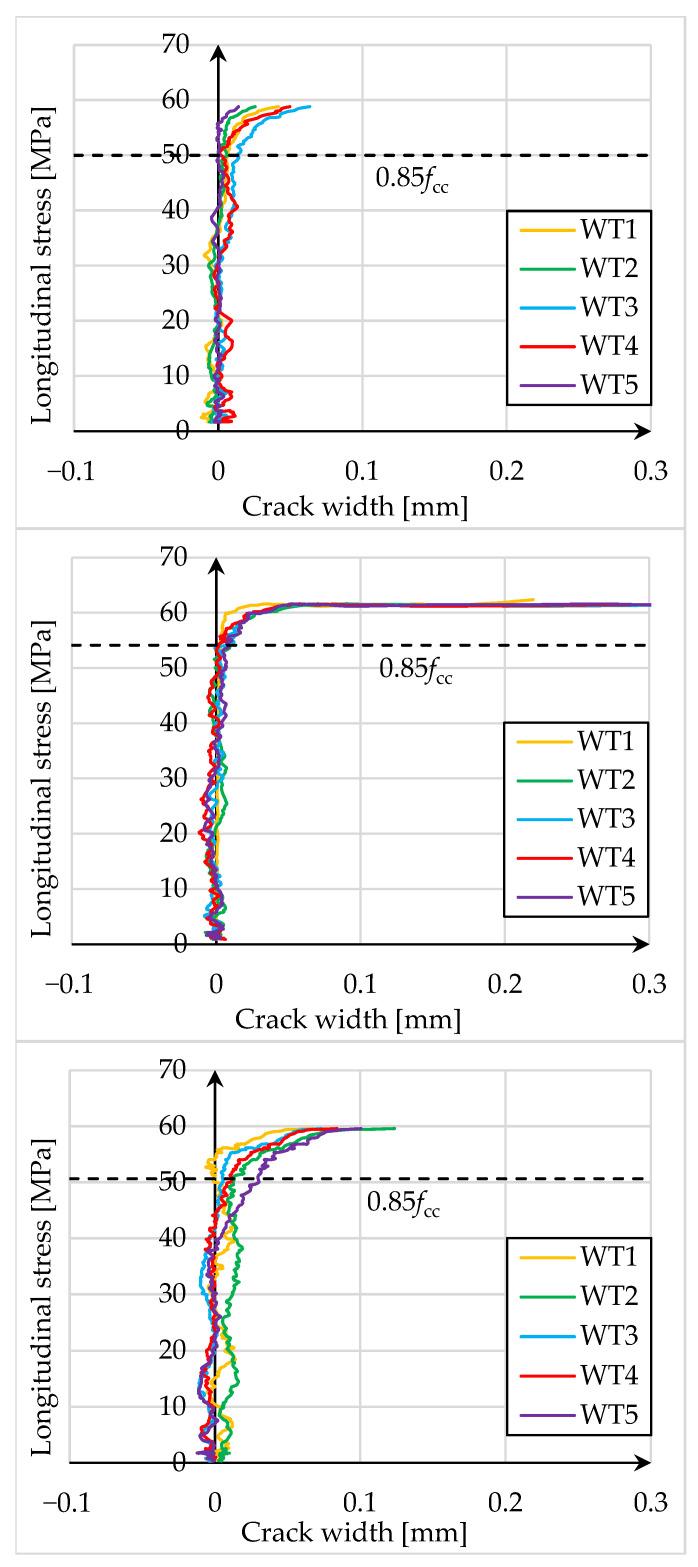
Longitudinal stress-crack width diagrams for specimens that were not pre-damaged, respectively: W3M01_0, W3M02_0, and W3M03_0.

**Figure 9:**
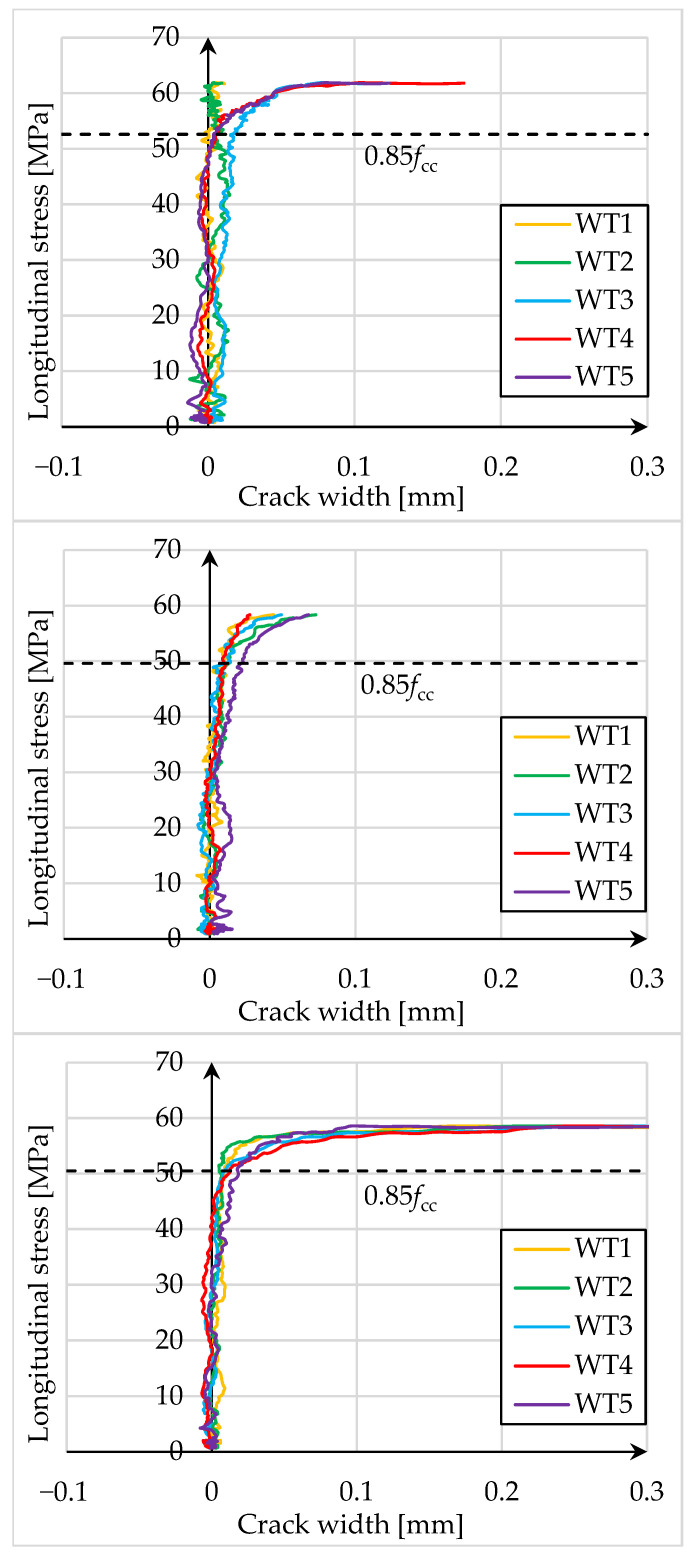
Longitudinal stress-crack width diagrams for pre-damaged specimens, respectively: W3M01_80, W3M02_80, and W3M03_80.

In addition, five virtual strain gauges (WT) were positioned in the center of the end line of the PBO mesh overlap, along a length equal to half the height of the cylinder, to measure the width of the crack. Figure 8 and Figure 9 show longitudinal stress in the element-crack width diagrams.

The presented test results show that the crack at the end of the PBO mesh overlap initiated at a stress of 85% of the strength of confined concrete. Subsequently, as the load increases, the crack expands rapidly, and the composite delaminates. The formation of the crack at the end of the overlap therefore occurs close to reaching the load capacity of the element and its failure.

#### 3.1.2. Eccentrically Compressed Specimens

**Figure 10:**
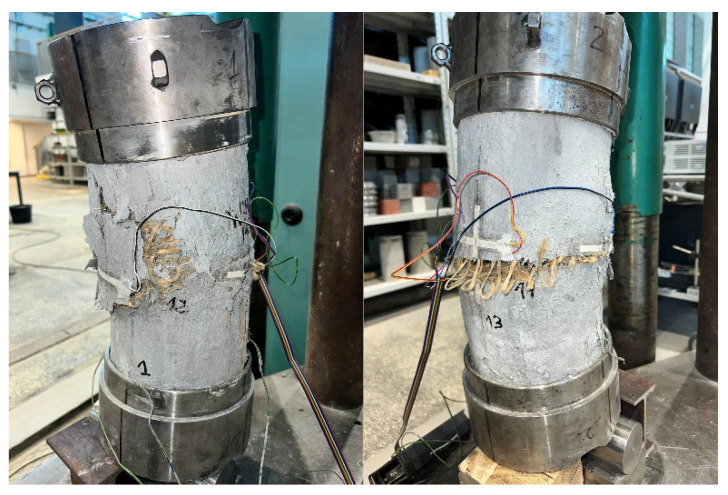
Failure images of eccentrically compressed confined specimens, not pre-damaged.

**Figure 11:**
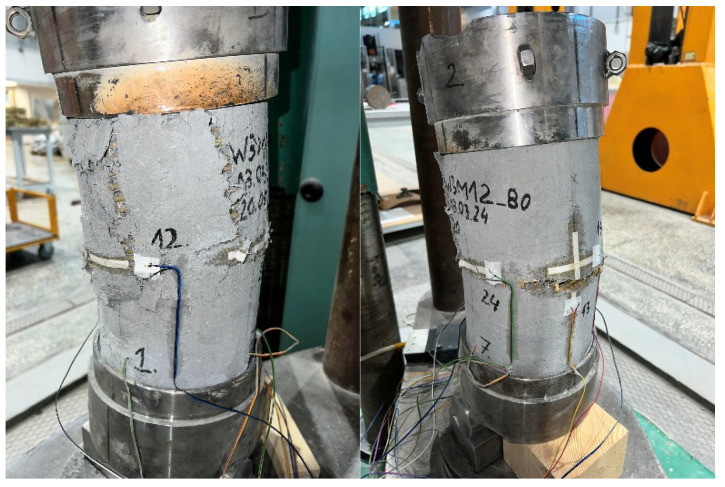
Failure images of eccentrically compressed confined specimens, pre-damaged.

The failure of eccentrically compressed specimens did not occur as a result of full delamination of the composite. Nevertheless, a crack at the end of the mesh overlap was noted, indicating that delamination had been initiated. All strengthened eccentrically compressed specimens failed due to crushing of the concrete in the compression zone. Rupture of hoop fibers of the PBO mesh in the compression zone of each element was also noted. In the tension zone, however, slippage of vertical fibers was usually observed. Only in elements W3M13_0, W3M12_80, and W3M23_80 was there a rupture of vertical fibers in the tension zone. Figure 10 and Figure 11 show sample images of the failure of strengthened eccentrically compressed specimens.

### 3.2. Load-Bearing Capacity and Deformability

#### 3.2.1. Axially Compressed Specimens

**Figure 12:**
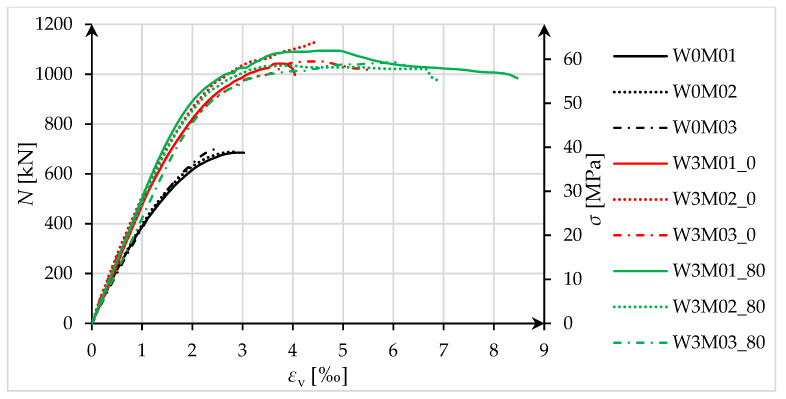
Force/stress–strain diagrams for axially compressed specimens.

The basis for analyzing the load-bearing capacity and deformability of compressed elements consists of the force/stress–strain relationships. Figure 12 shows force/stress–strain diagrams for axially compressed unstrengthened specimens (W0M0n), as well as for specimens that were not pre-damaged (W3M0_0) and pre-damaged specimens (W3M0_80).

Strengthened elements, both those that were not pre-damaged (red lines) and pre-damaged (green lines), achieved practically the same load-bearing capacity gains, which averaged 55% for specimens that were not pre-damaged and 53% for pre-damaged specimens.

The deformability was clearly higher for the pre-damaged specimens. The average increase in longitudinal strain at maximum force *ε*_cc_ in W3M0_80 specimens, relative to W3M0_0 specimens, was 16%. For ultimate strain *ε*_ccu_, the increase was 55%. The hoop strains *ε*_fl_ at maximum force in the strengthened specimens averaged 4.161‰ for specimens that were not pre-damaged and 4.567‰ for pre-damaged specimens. The difference did not exceed 10% and can therefore be considered negligibly small.

#### 3.2.2. Eccentrically Compressed Specimens

**Figure 13:**
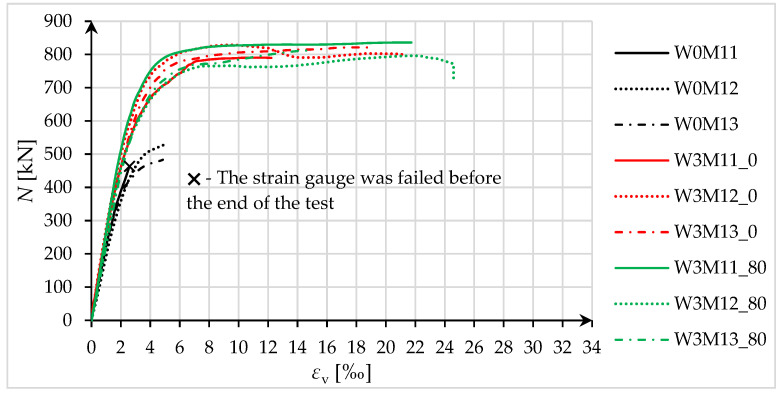
Force–strain diagrams for eccentrically compressed specimens, for eccentricity No. 1.

**Figure 14:**
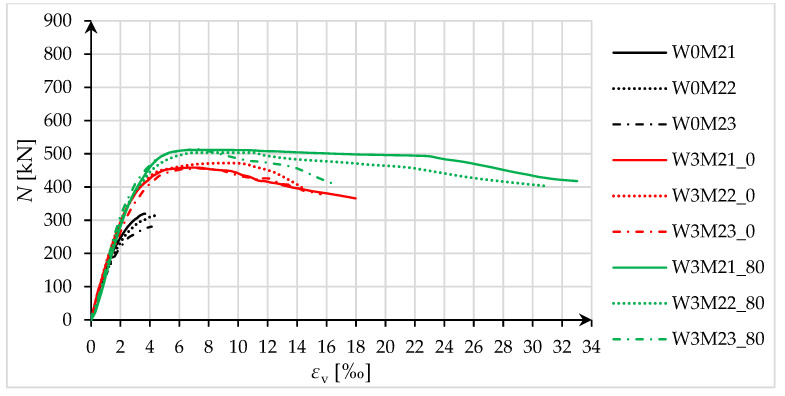
Force–strain diagrams for eccentrically compressed specimens, for eccentricity No. 2.

Specimens that were subjected to eccentric compression tests were analyzed in terms of load-bearing capacity and deformability with respect to the extreme compression zone. The force–strain diagrams are shown in Figure 13 and Figure 14.

For specimens compressed by force at eccentricity No. 1 (small eccentricity), with the force at the boundary of the core of the concrete section, there was no clear effect of pre-damage on the load-bearing capacity and deformability of the tested specimens (Figure 13). The average increase in load-bearing capacity for both W3M1_0 and W3M1_80 specimens was 55%. In contrast, the specimens subjected to pre-damage showed a higher load-bearing capacity than the specimens that were not pre-damaged in the case of eccentricity No. 2 (Figure 14). The average increase in load-bearing capacity for W3M2_0 specimens was 52%, while for W3M2_80 specimens, it was 67%.

Regarding longitudinal strains (in the extreme compression zone), an increase in strains at maximum force was observed (as in the case of axially compressed specimens) in the pre-damaged specimens, relative to the strengthened specimens that were not pre-damaged. The average increase in strain at maximum force *ε*_cc_ in W3M1_80 specimens, relative to W3M1_0 specimens, was 47%. For W3M2_80 specimens relative to W3M2_0, it was 13%. For ultimate strains *ε*_ccu_, the average increases in strain were, respectively, 18% and 67%. The hoop strains (in the extreme compression zone) at the maximum force in the specimens that were not pre-damaged and pre-damaged specimens were, on average, 4.620‰ and 4.809‰ (for eccentricity No. 2). For eccentricity No. 1, hoop strains were recorded only for specimens that were not pre-damaged (in pre-damaged specimens, strain gauges failed before the failure force was reached), and their average value was 10.005‰. Based on the measurements of hoop strains for eccentricity No. 2, the difference in the measured values is negligibly small, as it is less than 5%.

### 3.3. Ductility

The ductility of specimens was analyzed using two methods: first, based on toughness (Equation (1)) to achieve a maximum force in the element; and second, based on the ratio of ultimate strain to strain at maximum force *ε*_ccu_/*ε*_cc_. In the first method, ductility relative to reference specimens was determined using the comparative ductility index *C* (Equation (2) [22]). If the value of the index is equal to 1, then the ductility of the specimen under consideration is equal to that of the reference specimen. An index value greater than 1 indicates higher ductility, while a value less than 1 indicates lower ductility. This method is limited to comparing force/stress–strain relationships of similar shape. Additionally, it does not account for post-failure behavior.

(1) $$U_{T}=\int_{0}^{\varepsilon_{\mathrm{cc}}}N\left(\varepsilon_{v}\right)\;d\varepsilon_{v},$$

(2) $$C=\frac{U_{\mathrm{Tcc}}\cdot N_{c0m}^{2}}{U_{T0m}\cdot N_{\mathrm{cc}}^{2}},$$

where

*U*_T_—toughness;

*U*_Tcc_—toughness at maximum force for strengthened specimen;

*U*_T0m_—average toughness at maximum force for unstrengthened specimens;

*N*—compressive force;

*ε*_v_—vertical strain (in extreme compression zone for eccentrically compressed specimens).

#### 3.3.1. Axially Compressed Specimens

The ductility results for axially compressed specimens, using the two methods, are reported in Table 5.

An increase in ductility was recorded for both specimens that were not pre-damaged (except for one sample—W3M01_0) and pre-damaged specimens. The average values of the ductility index *C* were 1.07 for the first group and 1.34 for the second group. This shows that higher ductility (up to the maximum force) was obtained for pre-damaged specimens. Regarding the ratio *ε*_ccu_/*ε*_cc_, higher ductility was also obtained for pre-damaged strengthened specimens (*ε*_ccu_/*ε*_cc_ = 1.52) than for specimens that were not pre-damaged (*ε*_ccu_/*ε*_cc_ = 1.09).

#### 3.3.2. Eccentrically Compressed Specimens

The ductility results for eccentrically compressed specimens, using the two methods, are reported in Table 6 (for eccentricity No. 1) and Table 7 (for eccentricity No. 2).

For eccentrically compressed specimens, it should be noted that the ductility index *C* represents the behavior of specimens up to the maximum force. Therefore, when evaluating ductility based on Figure 13 and Figure 14, it is also necessary to consider the ultimate strains, because they are often much greater than the strains at maximum force, especially for eccentricity No. 2. Nevertheless, considering the analysis carried out using the *C* index, as in the case of axially compressed specimens, an increase in ductility due to strengthening was obtained for both specimens that were not pre-damaged, and pre-damaged specimens. The average values of the ductility index *C* for the respective groups of specimens were 2.07 and 3.20 (eccentricity No. 1), and 1.42 and 1.47 (eccentricity No. 2). Furthermore, the *ε*_ccu_/*ε*_cc_ ratios were 1.42 and 1.05 (eccentricity No. 1), and 2.11 and 3.02 (eccentricity No. 2).

## 4. Conclusions

The study of the effect of pre-damage on the behavior of concrete compression members strengthened with PBO-FRCM composite yielded the following conclusions:

- Based on the failure modes of the axially compressed specimens (Figure 4 and Figure 5), the hoop strain maps (Figure 6 and Figure 7), and the graphs of the stress-crack width relationship, it can be concluded that no clear influence of pre-damage on the failure mechanism was observed in the conducted tests.
- There was no clear effect of pre-damage on the failure mechanism of eccentrically compressed specimens, as shown in Figure 10 and Figure 11.
- The pre-damage effect did not affect the load-bearing capacity of axially compressed and eccentrically compressed specimens with eccentricity No. 1. However, for specimens with eccentricity No. 2, it resulted in a slight increase in load-bearing capacity.
- The longitudinal deformability was clearly higher for the pre-damaged specimens, which was most likely due to the degradation of the concrete structure. This degradation caused an increase in the transverse deformation of the concrete at the same stress level and consequently resulted in earlier activation of the strengthening system.
- There was no clear effect of pre-damage on the transverse deformability of strengthened specimens.
- Both axially and eccentrically compressed specimens subjected to pre-damage were more ductile than specimens that were not pre-damaged, which indicates a positive effect of pre-damage in this regard.

It should be noted that this study employed a relatively high external strengthening reinforcement ratio (based on [22]), which could have significantly influenced the positive aspects of the pre-damage effect. Furthermore, a relatively low pre-damage level was used, because concrete elements were tested (without steel reinforcement), which failed rapidly.

The conducted study focuses on the behavior of the concrete cross-section after strengthening; the effects of slenderness and reinforcement were not considered. An extension of the study to include reinforced concrete columns is planned for the future.

## Figures and Tables

**Table 1:** Concrete mix proportions.

Component	Amount (kg/m^3^)
CEM II 32.5R	404
Sand 0–2 mm	627
Gravel 2–8 mm	537
Gravel 8–16 mm	627
Water	175

**Table 2:** Parameters of PBO-FRCM components [23].

Parameter	Value
PBO-MESH 70/18	
Tensile strength of fibers (MPa)	5800
Young’s modulus of fibers (GPa)	270
Ultimate strain for fibers (%)	2.5
Equivalent thickness of mesh in warp (mm)	0.045
Equivalent thickness of mesh in weft (mm)	0.012
MX-PBO Concrete	
Compressive strength after 28 days (MPa)	≥40
Flexural tensile strength after 28 days (MPa)	≥4
Young’s modulus after 28 days (GPa)	≥15

**Table 3:** Types of specimens.

Type of Specimen	Designation	Strain Gauges Arrangement	
		Under the composite	On the composite
Axially compressed, unconfined	W0M0n	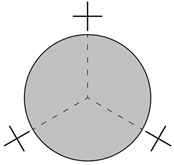	N/A
Axially compressed, confined	W3M0n	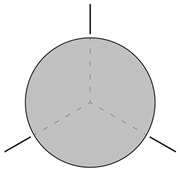	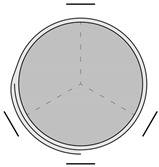
Eccentrically compressed, unconfined	W0M1n ^1^/W0M2n ^2^	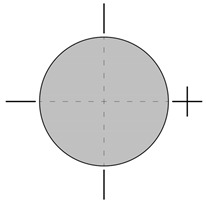	N/A
Eccentrically compressed, confined	W3M1n ^1^/W3M2n ^2^	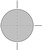	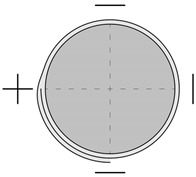

^1^ Eccentricity No. 1 equal to 1/8 of diameter (18.75 mm); ^2^ Eccentricity No. 2 equal to 1/4 of diameter (37.50 mm); n—number of specimen (three samples for each type of specimen); the right zone of the cross-section of eccentrically compressed elements is the extreme compressed zone; N/A—not applicable.

**Table 4:** Test results.

Specimen	*N*_cc_ (kN)	*M*_cc_ (kNm)	*f*_cc_ (MPa)	*N*_cc_/*N*_c0m_ (-)	*ε*_cc_ (‰)	*ε*_cc_/*ε*_c0m_ (-)	*ε*_ccu_ (‰)	*ε*_fl_ (‰)	*C* (-)
W0M01	685.26	0	38.78	N/A	2.943	N/A	3.026	N/A	N/A
W0M02	688.46	0	38.96	N/A	2.830	N/A	2.903	N/A	N/A
W0M03	699.12	0	39.56	N/A	2.459	N/A	2.459	N/A	N/A
W3M01_0	1042.52	0	58.99	1.51	3.789	1.38	4.039	3.465	0.94
W3M02_0	1126.58	0	63.75	1.63	4.422	1.61	4.422	-	1.07
W3M03_0	1051.39	0	59.50	1.52	4.579	1.67	5.489	4.858	1.21
W3M01_80	1094.33	0	61.93	1.58	4.852	1.77	8.465	4.758	1.28
W3M02_80	1034.88	0	58.56	1.50	3.919	1.43	6.928	4.376	1.03
W3M03_80	1048.36	0	59.32	1.52	6.037	2.20	6.240	-	1.72
W0M11	557.30	10.45	N/A	N/A	-	N/A	-	N/A	N/A
W0M12	532.48	9.98	N/A	N/A	5.170	N/A	5.170	N/A	N/A
W0M13	483.18	9.06	N/A	N/A	4.849	N/A	4.849	N/A	N/A
W3M11_0	790.39	14.82	N/A	1.51	11.471	2.29	12.219	13.043	1.81
W3M12_0	828.65	15.54	N/A	1.58	9.781	1.95	21.156	4.714	1.44
W3M13_0	821.12	15.40	N/A	1.57	18.294	3.65	18.861	12.258	2.98
W3M11_80	835.98	15.67	N/A	1.59	21.728	4.34	21.728	-	3.60
W3M12_80	796.09	14.93	N/A	1.52	21.793	4.35	24.596	-	3.68
W3M13_80	811.76	15.22	N/A	1.55	14.734	2.94	15.091	-	2.30
W0M21	319.66	11.99	N/A	N/A	3.678	N/A	3.678	N/A	N/A
W0M22	313.83	11.77	N/A	N/A	4.401	N/A	4.401	N/A	N/A
W0M23	280.77	10.53	N/A	N/A	4.117	N/A	4.117	N/A	N/A
W3M21_0	458.49	17.19	N/A	1.50	6.827	1.68	17.963	4.002	1.23
W3M22_0	471.99	17.70	N/A	1.55	9.309	2.29	14.445	6.641	1.75
W3M23_0	455.94	17.10	N/A	1.50	7.253	1.78	15.599	3.216	1.29
W3M21_80	511.47	19.18	N/A	1.68	8.640	2.13	33.018	4.796	1.43
W3M22_80	503.14	18.87	N/A	1.65	10.440	2.57	30.964	5.641	1.85
W3M23_80	513.76	19.27	N/A	1.69	7.239	1.78	16.361	3.990	1.15

*N*_cc_—failure force; *M*_cc_—bending moment corresponding to *N*_cc_; *f*_cc_—compressive strength; *N*_c0m_—average failure force for unstrengthened specimens; *ε*_cc_—vertical strain (in extreme compression zone for eccentrically compressed specimens) at maximum force; *ε*_c0m_—average vertical strain (in extreme compression zone for eccentrically compressed specimens) at maximum force for unstrengthened specimens; *ε*_ccu_—ultimate vertical strain (in extreme compression zone for eccentrically compressed specimens); *ε*_fl_—hoop strain in composite at maximum force; *C*—ductility index (described in Section 3.3); the suffixes “_0” and “_80” indicate, respectively, pre-damage levels of 0% and 80% of the average failure force determined from the reference specimens. N/A—not applicable.

**Table 5:** Ductility of axially compressed specimens.

Specimen	*U*_T_ (J/m)	*f*_cc_ (MPa)	*N*_cc_ (kN)	*C* (-)	*ε*_ccu_/*ε*_cc_ (-)
W0M01	1344.94	38.78	685.26	N/A	1.03
W0M02	1291.00	38.96	688.46	N/A	1.03
W0M03	1032.80	39.56	699.12	N/A	1.00
Avg.	1222.92		690.95		1.02
W3M01_0	2619.62	58.99	1042.52	0.94	1.07
W3M02_0	3468.34	63.75	1126.58	1.07	1.00
W3M03_0	3439.68	59.50	1051.39	1.21	1.20
Avg.				1.07	1.09
W3M01_80	3937.83	61.93	1094.33	1.28	1.74
W3M02_80	2821.17	58.56	1034.88	1.03	1.77
W3M03_80	4841.74	59.32	1048.36	1.72	1.03
Avg.				1.34	1.52

N/A—not applicable.

**Table 6:** Ductility of eccentrically compressed specimens, eccentricity No. 1.

Specimen	*U*_T_ (J/m)	*N*_cc_ (kN)	*C* (-)	*ε*_ccu_/*ε*_cc_ (-)
W0M11	-	557.30	N/A	-
W0M12	1892.41	532.48	N/A	1.00
W0M13	1699.81	483.18	N/A	1.00
Avg.	1796.11	524.32		1.00
W3M11_0	7368.26	790.39	1.81	1.07
W3M12_0	6466.39	828.65	1.44	2.16
W3M13_0	13,108.11	821.12	2.98	1.03
Avg.			2.07	1.42
W3M11_80	16,438.16	835.98	3.60	1.00
W3M12_80	15,253.00	796.09	3.68	1.13
W3M13_80	9921.10	811.76	2.30	1.02
Avg.			3.20	1.05

N/A—not applicable.

**Table 7:** Ductility of eccentrically compressed specimens, eccentricity No. 2.

Specimen	*U*_T_ (J/m)	*N*_cc_ (kN)	*C* (-)	*ε*_ccu_/*ε*_cc_ (-)
W0M21	774.37	319.66	N/A	1.00
W0M22	944.51	313.83	N/A	1.00
W0M23	794.94	280.77	N/A	1.00
Avg.	837.94	304.76		1.00
W3M21_0	2336.39	458.49	1.23	2.63
W3M22_0	3521.35	471.99	1.75	1.55
W3M23_0	2420.72	455.94	1.29	2.15
Avg.			1.42	2.11
W3M21_80	3364.59	511.47	1.43	3.82
W3M22_80	4225.10	503.14	1.85	2.97
W3M23_80	2733.42	513.76	1.15	2.26
Avg.			1.47	3.02

N/A—not applicable.

## Data Availability

The original contributions presented in this study are included in the article. Further inquiries can be directed to the corresponding author.
